# The lived experiences of resilience in Iranian adolescents living in residential care facilities: A hermeneutic phenomenological study

**DOI:** 10.3402/qhw.v11.30485

**Published:** 2016-03-01

**Authors:** Manijeh Nourian, Farahnaz Mohammadi Shahboulaghi, Kian Nourozi Tabrizi, Maryam Rassouli, Akbar Biglarrian

**Affiliations:** 1 Nursing Department, University of Social Welfare and Rehabilitation Sciences, Tehran, Iran; 2 Social Determinants of Health Research Center, Nursing Department, University of Social Welfare and Rehabilitation Sciences, Tehran, Iran; 3 School of Nursing and Midwifery, Shahid Beheshti University of Medical Sciences, Tehran, Iran; 4 Department of Biostatistics, University of Social Welfare and Rehabilitation Sciences, Tehran, Iran

**Keywords:** Adolescents, hermeneutic, Iran, residential care facilities, resilience

## Abstract

**Background:**

Resilience is one of the main factors affecting human health, and perceiving its meaning for high-risk adolescents is of particular importance in initiating preventive measures and providing resilience care.

**Objectives:**

This qualitative study was conducted to explain the meaning of resilience in the lived experiences of Iranian adolescents living in governmental residential care facilities.

**Materials and methods:**

This study was conducted using the hermeneutic phenomenological method. Semi-structured interviews were conducted with eight adolescents aged 13–17 living in governmental residential care facilities of Tehran province affiliated to the Welfare Organization of Iran who articulated their experiences of resilience. Sampling lasted from May 2014 to July 2015 and continued until new themes were no longer emerging. The researchers analyzed the verbatim transcripts using Van Manen's six-step method of phenomenology.

**Results:**

The themes obtained in this study included “going through life's hardships,” “aspiring for achievement,” “self-protection,” “self-reliance,” and “spirituality.”

**Conclusion:**

Our study indicates that the meaning of resilience coexists with self-reliance in adolescents’ lived experiences. Adolescents look forward to a better future. They always trust God in the face of difficulties and experience resilience by keeping themselves physically and mentally away from difficulties. Adverse and bitter experiences of the past positively affected their positive view on life and its difficulties and also their resilience. The five themes that emerged from the findings describe the results in detail. The findings of this study enable nurses, health administrators, and healthcare providers working with adolescents to help this vulnerable group cope better with their stressful life conditions and improve their health through increasing their capacity for resilience.

The number of adolescents sent to out-of-home care is increasing throughout the world and 8 million children are estimated to currently be residing in these facilities (Bos & Zeanah, 2011). Previous studies have shown that experiences of children in care facilities do not indicate a necessarily protective or supportive nature, and this vulnerable group of is exposed to a variety of stressors and problems (Bos & Zeanah, 2011; Drapeau, Saint-Jacques, Lepine, Bégin, & Bernard, 2007; Ellis, Fisher, & Zaharie, 2004). These problems exert negative effects on their health and put them at a much greater risk of developmental, social, and behavioral disorders (Garmezy, 1993; Marquis & Flynn, 2009; Schowalter & Costello, 1989); mental disorders; academic challenges; low self-confidence; and communication problems (Barnsley, 2011). Studies suggest that the problems experienced in adolescence and the health disorders developed in life are closely linked (Aarons et al., 2010; Barnsley, 2011; Barth et al., 2007; Jozefiak et al., 2016; Leon, Ragsdale, Miller, & Spacarelli, 2008). Living in residential care facilities increases the likelihood of engaging in high-risk behaviors and causing harms in the adolescents and thus threatens the health and safety of the community as this group leaves the facilities and enters the community.

As for preventive measures that can help resist these outcomes, experts emphasize the reinforcement of protective elements such as resilience instead of a focus on eliminating the existing risk factors (Hunter, 2001). Resilience is a capacity that is highly influenced by the environment in which one lives and is crucial to the ability to cope with adversity and to survive it (Wagnild &Young, 1993). It has positive effects on stress levels, leads to a higher quality of life, and promotes overall health (Chia & Lee, 2015).

The majority of studies conducted on this topic have been carried out in English-speaking countries and Europe and suggest that resilience is a multi-dimensional feature that varies with cultural origin (Alizadeh, 2013; Connor & Davidson, 2003; Harvey & Delfabbro, 2004). As an example, Werner (1995) defined resilience as good developmental outcomes and sustained competence despite the presence of stress and risk factors. Rutter (2006) called resilience an interactive concept that is associated with relative resistance to risky environmental experiences or overcoming troubles and stress. Polk (1997) defined resilience as the ability and capacity to change difficult conditions to experiences that lead to the growth and development of an individual.

Studies have often taken a quantitative approach in examining the factors influencing resilience. The limited number of qualitative studies conducted on this topic tends to focus on the sources of resilience in adolescents living in care facilities and the factors affecting them. The differences in qualitative research findings further endorse the fact that resilience has different definitions and sources in different communities, cultures, and supportive care systems (Bell & Romano, 2015; Chavarria & Johnson, 2014; Hass, Allen, & Amoah, 2014; Pienaar, Swanepoel, Van Rensburg, & Heunis, 2011).

However, the lack of a clear definition on resilience (Luthar, Cicchetti, & Becker, 2000; Kaplan, 2005; Masten, 2007) is the first and foremost obstacle in the initiation of preventive and resilience-based interventions and in improving the health status in this high-risk group of adolescents.

Given that resilience is a complex, multi-dimensional, and context-based construct, the researcher used the interpretative phenomenological method for exploring its meaning. Understanding the concept of resilience in the Iranian context enables nurses, caregivers, health decision-makers, and directors of care facilities to help these vulnerable adolescents cope better with their demanding life conditions and improve their health through a set of preventive measures and an improved resilience.

Despite the importance of resilience for high-risk groups of adolescents living in adversity (Luthar, Cicchetti, & Becker, 2000), few studies have examined this concept in adolescents residing in residential care facilities in Iran.

The present research is perhaps the first effort in Iran for exploring the meaning of resilience as manifested by the adolescents residing in residential care facilities.

## Materials and methods

The present hermeneutic phenomenological study was conducted on the population of Iranian adolescents residing in 15 governmental residential care facilities (also called pseudo-family centers) using Van Manen's six-step method of phenomenology. This method was applied within the following six steps: (1) turning to the nature of the lived experience; (2) investigating the experience as we live it; (3) reflecting on the essential themes that characterize the phenomenon; (4) describing the phenomenon through the art of writing and rewriting; (5) maintaining a strong and oriented relation to the phenomenon; and (6) balancing the research context by considering parts and whole. Van Manen's method provides a guideline for researchers and is useful for conducting hermeneutic phenomenological studies in practice (Van Manen, 2001).

In the first step, researchers wrote about the means of creating personal and professional motivation for studying the phenomenon of resilience and documented their previous assumptions, for example, about their definition of resilience and their ways of viewing the phenomenon. These pre-assumptions were the result of working with children as a pediatric nurse (the first and the fourth authors) and as a researcher on adolescents and their difficulties (the fourth author). The second author's pre-assumptions result from the research on out-of-home care and also prominence in conducting qualitative studies. The third author had experiences in working with adolescents living in support centers, which could lead to his knowledge about adolescents’ difficulties and the importance of comprehending the meaning of resilience. The researchers’ knowledge in the present study was mostly based on the fact that the adolescents living in support centers are exposed to more adversities in their life and experience more resilience. Difficult living conditions in residential centers pave the way for resilience, and this group of adolescents experience resilience differently from those living with their families. Maintaining openness to the phenomenon, researchers put aside their pre-understanding before and during interviews and refer to them later in the process of analysis (Van Manen, 2001).

### Sampling and study setting

The research question was developed according to the first and second steps of Van Manen's method, and participants were selected through purposive sampling from a population of adolescents aged 13–17 with no or poor supervision. In Iran, when all the efforts to return unsupervised children to their original or substitute families are futile, governmental residential care facilities take them in for reasons such as the death or absence of one or both of the parents or their divorce. These facilities are single-gendered and adopt a psychotherapeutic perspective in raising these children.

All the study participants had been living in the governmental residential care facilities examined under the supervision of the Welfare Organization of Tehran Province at the time of interview for at least 3 years and were able to speak Persian. Adolescents who had a history of mental, developmental, seizure disorders, and physical or motor disabilities according to their health records were excluded from the study. Participants who had high levels of resilience according to the Persian version of the Wagnild and Young Resilience Scale (Wagnild & Young, 1993) were invited to be interviewed.

Coordinations were made with the technical directors, psychologists, or social workers of the centers. The researcher introduced herself to the eligible candidates and asked them to sign an informed consent form instructing on the study objectives, participants’ right to withdraw from the study, and the confidentiality of their data. A demographic questionnaire was used to collect participants’ name, age, gender, date of birth, duration of time spent in the center, and school grade levels. Participants then filled out the Resilience Scale under the supervision of the researcher and after they had received instructions on how to complete it. The eligible adolescents were then interviewed in the afternoon after their school time. Again, informed consent was obtained before conducting any interviews. Samples were selected through maximum variation sampling, which lasted from May 2014 to July 2015 and continued until new themes were no longer emerging.

### Ethical considerations

The research project was approved by the Medical Ethics Committee under the code uswr.rec.1393.211 for compliance with the principles of research ethics and was part of a nursing PhD thesis approved by the University of Social Welfare and Rehabilitation Sciences, Tehran, Iran.

To observe the ethical standards codes of the Helsinki Declaration (2001), the researcher informed all participants of the objectives and significance of the study. Participants and their guardians (technical directors) submitted their informed consent to participate in the study and also consented to having their statements tape-recorded during the interviews. The principle of confidentiality of information was observed and the adolescents were assured of not mentioning their names in any stage of the study. Issues leading to the recognition of interviewees were not mentioned. All documents related to the participants were kept in a special folder in a safe place. The right to resign was reserved with no restrictions for the participants. Whenever an adolescent got tired or was not willing to continue, the interview was postponed to the next turn. (One of the adolescents was interviewed in two stages.) They were treated with respect at all the stages. They were also reassured that their condition would by no means be affected by their statements and all their remarks would remain confidential.

### Interview process

The interviews started with a general conversation about the problems the adolescents were faced with and continued with open-ended questions, including “What does the word *resiliece* make you think of? Have you ever encountered a problem that has made you feel defeated but then managed to overcome it? How did you feel in the circumstances? How would you describe this experience?” To allow for a quieter and less crowded setting, the interviews were conducted in the living room (seven interviews) or the dining hall (one interview) of the centers as per the adolescents’ liking. Given the adolescents’ enthusiastic expression of their experiences of the concept, the researcher kept silent throughout the sessions in the attempt to achieve a deeper meaning of resilience and posed only contemplative questions. The researcher recorded the adolescents’ non-verbal behaviors at the end of each interview. The interviews were semi-structured, lasted between 38 and 63 min and were conducted by the first author who is a professional pediatric nurse and has experience of work with adolescents.

### Analysis and interpretation

The Van Manen view (2001) was used to explore and analyze the concept of resilience from an interpretive point of view.

A holistic and line-by-line approach was used to separate the thematic statements made in the interviews. After reading each interview several times and proceeding with its verbatim transcription, the researcher wrote down her own general understanding of the interviews in a few paragraphs. Then, the researcher re-read the interview text line by line and separated the phrases and sentences that were relevant to resilience and transformed them into the initial thematic statements. The researcher carried out a constant comparison of the initial thematic statements extracted from all the new interviews and then classified them into subthemes based on their similarities. The initial common themes gradually emerged as the study progressed, and the focus of all subsequent interviews became then to compare these themes and combine the similar ones. The researcher kept going back and forth between the general perception and the initial thematic statements during the interviews. The first and second authors engaged in a hermeneutic dialogue about the subject throughout the process of interviewing. The research team then proceeded to examining each single theme emerged and separated the essential ones through the method of free variation in imagination and by comparing the themes with other phenomenological texts. The researcher used the notes written during the analysis stage and after the interviews to provide examples of participants’ statements that helped formulate the phenomenological text constituting the research findings.

### Rigor

For achieving credibility, the researcher was frequently and closely in touch with the adolescents for a period of 12 months and used methods such as taking notes during and after the interviews and the careful observation of the adolescents’ behaviors and their non-verbal messages to better explore the meaning of resilience. The initial thematic statements and subthemes and the original text of the interviews were presented during a meeting to two of the participants, who then approved the researcher's perceptions. The findings were discussed within the research team during the process of analysis. Dependability and confirmability were maintained through the use of documentation during the study. The initial thematic statements and examples of the subthemes and excerpts extracted from the interview texts were presented for each theme to five faculty members in psychiatric nursing, psychology, and pediatrics who were experts in phenomenological research during the process of analysis and their views were collected. Two sessions (progression report) were held with the university research committee. For achieving transferability, the researcher attempted to provide a detailed and complete description of the process of research to facilitate the future assessment of the study and also participants were selected through maximum variation sampling.

## Results

As shown in Table I, participants consisted of eight adolescents residing in different residential care facilities for a duration of 3–15 years (8.75±4.39), with high levels of resilience and aged 13–17 (14.87±1.67). A total of 511 initial thematic statements were extracted from the data, forming five themes (Table II).

**Table I:** Participants’ characteristics.

Participant	Gender	Age (in year)	Education	Resilience score (level)	Duration of time residing in residential care facilities (in year)
1	Male	17	Middle school	113 (high)	11
2	Female	14	Elementary school	100 (high)	8
3	Male	16	High school diploma	110 (high)	15
4	Male	13	Middle school	105 (high)	4
5	Female	15	High school diploma	102 (high)	14
6	Male	14	Middle school	108 (high)	6
7	Male	17	High school diploma	101 (high)	9
8	Female	13	Elementary school	100 (high)	3

**Table II:** The subthemes and main themes emerged from the data.

Initial subtheme	Subtheme	Main theme
Resistance	Endurance	Going through life's hardships
Tolerance		
Learning from difficult past experiences	Finding strength in difficulties	
Growing amid the problems		
Maturing through helplessness		
Behavior rooted in comprehensive evaluations	Engineering the difficulties	
Seeking help from people with similar experiences		
Positive thinking	Optimism	Aspiring for achievement
Hopefulness		
Self-encouragement		
Making efforts	Meaningful life	
Having goals		
Trying to avoid drowning in the problems of life	–	Self-protection
Getting away from problems		
Having a special bond with God	–	Spirituality
Relationship with oneself and the others		
Self-reliance	–	Self-reliance

### Going through life's hardships

Three subthemes emerged for this main theme, including “endurance,” “finding strength in difficulties,” and “engineering the difficulties.”

#### Endurance

One of the major subthemes discussed by all participants was “endurance,” which consists of two initial subthemes, including “resistance” and “tolerance.” All participants acknowledged that resilience meant not giving up and continuing to resist the problems, which require tolerance and refraining from obsessing over the problem. They endured their stressful life conditions out of the fear that their past difficulties may re-emerge and destroy their life once again. One participant stated, “… Resilience means to resist, when one tolerates or doesn't obsess over the problem, he won't fall and can stand on his own two feet; when you tolerate a problem and carry on, new problems won't emerge …” (P3).

#### Finding strength in difficulties

This subtheme was common in the statements of all participants and has three initial subthemes, including “learning from difficult past experiences,” “growing amid the problems,” and “maturing through helplessness.” Participants led stressful lives filled with problems and had annoying thoughts about the hardships and bitterness of their past life; however, the majority of them said that their difficult past experiences, the helplessness and continuous loneliness they felt as a result of those hardships, and the persisting limitations and problems they were faced with in these centers had positive effects on their tolerance of problems and resistance toward them. One of the adolescents said, “… I get very upset every time I remember the past and my parents’ quarrels, but the past is like a book. Honestly, we have many problems here too, but my past helps me find a way to deal with things and it has also increased my levels of tolerance. Both are very useful, they make us strong …” (P1).

Living in residential care facilities and dealing with frequent sources of stress contributed to the degree of resilience built in participants. One adolescent stated, “… Now I have learned that, actually, living *here* has taught me that, since problems tend to be constantly repeating themselves here, you realize that you shouldn't really get upset over anything, that you have to wait everything out …”(P7).

#### Engineering the difficulties

This subtheme consists of two initial subthemes, including “behavior rooted in comprehensive evaluations” and “seeking help from people with similar experiences.” Whenever the adolescents felt that their problem could be solved by themselves, they tried to examine all the possible solutions by contemplating and performing a comprehensive evaluation of the problem and through assessing their own capabilities; furthermore, they projected a balanced reaction to the problems, which was rooted in their difficult past experiences and what they had learnt from the experiences of their peers in the centers and knew that they had to deal with problems without getting entangled with them and by maintaining their current conditions. A 14-year-old adolescent said about his experiences, “… For example, I managed to save my sister from my mother, I knew her future would be damaged, and I couldn't stop thinking about it, so, for several nights, I kept thinking of every possible way through which I could solve the problem. Then I had to talk to a friend who lives here, for I couldn't share it with the center's authorities, and my friend told me that we had to call his uncle. We made some arrangements and finally managed to get Najla used to milk powder. I was finally relieved …” (P2).

### Aspiring for achievement

This theme has two subthemes, including “optimism” and “meaningful life,” which were repeated in many of the interviews in a lot of the thematic statements made.

#### Optimism

This subtheme consists of three initial subthemes, including “positive thinking,” “hopefulness,” and “self-encouragement.” A large number of participants tried to endure their hardships through positive thinking and by focusing on the positive aspects of life in residential care facilities and by pondering on the positive side of their problems. They tended to withstand the problems with a greater hope in the future and through self-encouragement. One participant said, “… With the problems I have, the fact that I don't have a mother (silence), and the other difficulties I have had to endure, well we have quite some problems here too and we get annoyed, but we wait them out. I know my future is bright. There are a lot of kids outside on the streets. We have it much better here …” (P6).

A number of participants discussed self-reproach and self-encouragement as factors helping them move forward, try to solve the problems, and hold positive inner dialogues. One of the adolescents said these dialogues, “At that point I was talking to myself, saying that it's enough and that I should do something. I encouraged myself, I told myself not to do that, that carelessness was enough, because I couldn't take it anymore” (P1).

#### Meaningful life

This subtheme consists of two initial subthemes, including “making efforts” and “having goals” A number of participants discussed the need for having a goal in life and for striving to achieve it, which leads to resisting problems and tolerating them. One of the adolescents said about the importance of this issue, “…Look, I know that, when someone wants to achieve something, he withstands the hardships better. When you fight and try, you gain at least something, and those things make you be able to tolerate the conditions better. Different things affect each other, in a way, it's like a circle …” (P3).

### Self-protection

This theme consists of two initial subthemes, including “trying to avoid drowning in the problems of life” and “getting away from problems.”

#### Trying to avoid drowning in the problems of life

Participants’ experiences indicated their efforts for keeping their current life conditions safe from the negative consequences of the problems with which they were dealing. A 13-year-old male adolescent said, “… You shouldn't drown yourself in your problems. You shouldn't let the problems overwhelm you. When you can't fix them, you should let go and not fall into their trap …” (P4).

#### Getting away from problems

Through avoiding the problem and distancing themselves from it, the adolescents dealt with the problem in a way that it did not make them lose their mental well-being and peacefulness and did not destabilize their current conditions.

Noting her difficult past experiences, another adolescent said of her attempts to protect herself against her problems, “… My parents were always fighting. Their fights were terrible, but I tried not to interfere so that I wouldn't get bummed. Now, if I have a problem here, I do the same thing. I won't dwell on it. I won't interfere and will just let go …” (P5).

### Spirituality

This theme consists of two subthemes, including “relationship with oneself and the others” and “having a special bond with God.”

#### Relationship with oneself and the others

In the face of difficulties, participants ensured through their relationships that they would have a system of support whenever necessary. They felt more peaceful this way and gained the composure required for dealing with the problems of life. One 15-year-old adolescent said of these relationships, “… A good relationship with friends provides you with a system of support; when you have a problem and you know that your friend is there, even if they don't really help you, you feel more composed and can manage to do something yourself …”(P5).

#### Having a special bond with God

Participants hoped and believed in the Divine Mercy whenever they faced difficulties; through their special bond with God, they attempted to keep their calm in the face of problems and resist in the shadow of this peace. Although the trust in God and the Infallible Imams could be discerned in the discussions with all participants, and although their sense of helplessness only exacerbated this trust, it was more intense and noticeable in the female adolescents and those who had lived in the centers for shorter periods of time and who were younger. A 13-year-old adolescent said, “… When there is a problem and you call on Imam Ali, you find a peace that helps you not make decisions in anger …” (P8).

### Self-reliance

In the experience of participants, resilience meant being reliant on oneself and doing everything within one's power to withstand the difficulties. Participants tried to establish strong stable relationships with the people around them, especially with their teachers and social workers; however, since they were mostly deprived of these relationships, they tended to rely on themselves for dealing with any problems that arose, and only in times of real emergency, when the problem was very serious and intolerable, they asked for help from a trusted person (often their peers in the center). One of the adolescents noted, “… I only count on myself. I do what I can. It's better not to count on others at all when you have problems. Others may even make the problem worse …” (P2).

## Discussion

The present research is one of the first qualitative phenomenological studies conducted to explore the meaning of resilience in the experience of adolescents living in residential care facilities.

The concepts that emerged from the present study have some differences and some similarities with the studies conducted on the same subject in other countries (Bell & Romano, 2015; Drapeau et al., 2007; Hass et al., 2014; Hunter & Chandler, 1999; Pienaar et al., 2011). The first theme that emerged from the adolescents’ experiences was “going through life's hardships,” that is, moving forward despite a difficult and stressful life, which constituted one of the major dimensions of resilience in the adolescents surveyed. Fearing the recurrence of past problems and the loss of their present conditions and influenced by feelings of loneliness caused by their difficult past experiences, the absence of emotional support and the lack of a stable relationship with the center's authorities and teachers, the adolescents were found to approach their problems with caution, to often stay away from the ones they could not handle (which was often the case), to tolerate them without preoccupying their mind with the matter, and to live with the hope of a better future upon leaving the center. They asked for the help of their peers and used the capacities they had acquired through their difficult past experiences to evaluate their problems from all aspects; they reflected on the problem at hand, evaluated their capabilities, and then showed a balanced reaction to the issue if they felt that they could properly handle it. It was very important to them to not allow a problem they were not fully capable of handling to worsen their life and thus tried to avoid such problems altogether. They avoided putting themselves physically or mentally in positions that led to the problem becoming bigger or having worse consequences. They thus took care of themselves and protected their present conditions and peace of mind.

Previous studies conducted on resilience in adolescents living in care facilities have not reported this theme in their findings (Hass et al., 2014; Henry, 1999; Pienaar et al., 2011; Shepherd, Reynolds, & Moran, 2010). The life conditions of Iranian adolescents residing in residential care facilities, which are themselves affected by the care models established or by economic, social, and cultural factors, appear to have affected the adolescents’ perception of resilience and the emergence of this theme.

The examined adolescents’ problem endurance can be said to be rooted in their own beliefs as well as in the dominant culture of Iran. Guided by this culture, the adolescents attributed their ability to withstand problems to their capacity for endurance. This subtheme is also manifested in Persian literature, particularly in the poetry of Saadi, “Treasure will not be gained without suffering/Morning will not emerge until the night is through,” and Hafiz, “Endure the night and day in hardship, Hafiz/Until you attain the object of your desire.”

Studies conducted on the concept of resilience in other stressful life conditions have reported similar themes; for example, one of the themes that emerged in a study on patients with chronic pain was “moving forward with one's life” (West, Buettner, Stewart, Foster, & Usher, 2012); in another study, participants defined resilience as choosing life and moving forward with it and carrying out a simple living (Edward, Welch, & Chater, 2009). However, in the present study, “going through life's hardships” appears to have a deeper meaning than just “moving forward with one's life.”

Although several studies have shown that severe difficulties and stressful experiences may lead to many problems through creating a negative perception of the world (Monson, Gradus, La Bash, Griffin, & Resick, 2009), in the present study, participants showed that their difficult past experiences and the subsequent sense of helplessness have made them stronger in the face of problems. Evidence suggests that people's ways of interpreting their experiences and making assumptions about the world affect their compatibility with problems. Becoming stronger through negative life events is associated with positive changes, such as changed priorities, improved self-efficiency, and higher spirituality (Barskova & Oesterreich, 2009). These changes increase the individual's power to withstand the problems and thus improve his compatibility with new conditions. In Samuels and Pryce's study (2008), participants stated that their mental suffering and difficult life experiences were a “source of strength” for them. On the other hand, the belief in becoming stronger through the endurance of hardship is rooted in the Iranian culture; in fact, Iranians use the term “tempered steel” in their routine conversations to imply a person who has endured a lot of hardship in life and has thus become stronger and more tempered.

“Aspiring for achievement” was another main theme that emerged in the present study. Influenced by their bitter past experiences, the adolescents tended to focus on the positive aspects of their lives in the center and also of their problems. They made their problems more bearable and experienced resilience through viewing their problems as more trivial than they actually were and through keeping their hopes of a better future and planning for when they would finally step outside the center.

Some studies have shown similar findings with respect to this aspect of resilience (Bell & Romano, 2015; Chavarria & Johnson, 2014; Pienaar et al., 2011). In Pienaar et al.'s study (2011), conducted on the meaning of resilience in adolescents with HIV living in institutional care facilities, optimism and a positive outlook on life were parts of the “internal sources of strength and individual capacity”, which are consistent with the subthemes of “optimism” and “positive thinking” that emerged in the present study. In addition, Pienaar et al.'s study proposed having a goal in life and hopefulness as sources of resilience and formed the theme of “interpersonal and problem-solving skills.” Chavarria and Johnson (2014) also discussed a positive outlook on life as a main theme, which is in a way consistent with the “positive thinking” subtheme of “aspiring for achievement.” For participants of the present study, having led difficult lives had not resulted in a positive outlook on oneself, rather on living in these centers and on the difficulties with which they were faced there, or, as in the study conducted by Chavarria and Johnson, on the services provided by these centers, their associated stresses and generally the future. In a study focusing on the education aspect of resilience, having a goal was reported as one of the main sources of support in care centers (Hass & Graydon, 2009). In a review study, Jackson, Firtko, and Edenborough (2007) argued that resilient individuals have the ability to look at the positive aspects and the potential benefits of a situation.

Participants manifested “self-encouragement” through projecting positive self-blame and reproaching themselves. Although researchers believe that encouragement is one of the factors associated with resilience, they did not report findings similar to the “self-encouragement” subtheme manifested in the present study in participants’ positive inner dialogues. Nevertheless, self-encouragement helps with increasing one's degree of resilience and is recommended to be further examined in future studies.

Participants showed that behaviors such as withdrawal, problem avoidance, not obsessing over the problem, and keeping silent were ways of protecting themselves from the problem and keeping their mental peace. The self-protection theme has been commonly discussed in studies on adolescents’ resilience. For instance, in the qualitative part of the study conducted by Hunter and Chandler (1999), high school adolescents living in poor areas stated that they kept themselves away from those they thought could harm them and protected themselves in the face of difficulties without trusting others and asking for their help. Some studies have discussed “distancing oneself from the source of danger” as one of the themes emerging from the experiences of adolescents living in care facilities of resilience, which is somewhat similar to the “trying to avoid drowning in the problems of life” initial subtheme that emerged in the present study (Drapeau et al., 2007). “Keeping away from abusers” was a theme extracted in a study by Henry (1999) on the experiences of resilient adolescents who had been abused in the past and had now been adopted by a new family.

Hunter (2001) extracted the subtheme of “self-protection” through his qualitative analysis of the role of culture in the degree of resilience in US adolescents. He concluded that adolescents with an adequate support system project a communicational form of resilience, while those not protected by their family or the society project a protective form of this quality, which involves avoiding others and protecting oneself. It seems that participants in this study show the self-protection form of resilience due to their apparent lack of support on the part of their caregivers and the absence of strong and stable relationships with those around them, especially with their caregivers and social workers.

“Spirituality” was one of the main themes that emerged in the present study. Participants’ hope in God's Mercy gave them peace; it helped them accept their conditions and their lives as God's will. The hope and trust in God and His grace were the biggest factors that helped participants achieve a certain degree of peace and positive adaptation in the face of hardship.

They hoped for a better future life by placing their trust in God and His grace and found the peace required for enduring their problems and for deciding on how to deal with them through establishing connections with their peers.

They called on the Imams when facing problems and asked for their help. This perspective on spirituality can be rooted in the Iranian and Islamic culture in which the adolescents lived. Asking for God's help and trusting in His grace and calling out the names of the Holy Imams in the face of problems are common phenomena in the Islamic Iranian society and are in fact rooted in the ancient Iranian culture. In a study conducted in Iran, the “trust in God” was one of the themes emerging from the experiences of adolescent girls living in residential care facilities of spiritual coping, who stated that the “trust in God” and “praying” were useful strategies for dealing with problems (Rassuli & Yaghmai, 2010). In the study conducted by Pienaar et al. (2011), the adolescents discussed “external support” as a main theme and stated that their systems of support derived from their relationships with friends, caregivers, professionals (teachers, psychologists, and nurses) and ultimately with God.

All the participants emphasized that they did not count on anyone's help in the face of difficulties and considered themselves independent and self-reliant; however, unlike the study conducted by Hass et al. (2014), their independence and self-reliance did not originate from having a sense of control over the environment and was rather due to the lack of trust in the sincere help of people around them, especially of the center's authorities, and also due to feelings of helplessness. However the subtheme of self-reliance and independence comprise another concept supported in other studies, such as in the one conducted by Hunter and Chandler (1999).

### Limitations

According to Van Manen (2001), an interview to achieve participants’ lived experiences is a priceless resource for the deep understanding of the phenomenon to be studied. In addition to interview, Van Manen introduced other methods such as a detailed observation as an indirect method of collecting information, which allows the researcher to be involved in the participant's world (Van Manen, 2001). Owing to the wonderful willingness of the adolescents living in residential care centers to express their experiences and feelings, the present study used only an oral interview as the main method to collect data. Given rules and regulations governing centers, a long and continued stay was not possible for the researcher to make observations as the second method of data collection, and the researcher (the first author) attempted to make notes of non-verbal messages of adolescents like tone, silence, and crying immediately after every interview. The notes along with the field notes at the data analysis stage created a deeper comprehension of the adolescents’ statements.

The results of qualitative studies cannot be generalized; however, sampling purposively and recording the research process and the decisions made in this process, the researchers tried to make it possible for other researchers to follow the research process and therefore increase the probability transferring the findings of the present study to similar situations like non-governmental residential centers.

According to the centers’ rules and regulations, the center authorities were in charge of determining the time and place of further interviews, and holding the interview sessions outside the centers was not a possibility; these limitations may have affected the adolescents’ tendency for giving a full description of their experiences.

## Conclusion

The findings obtained from adolescents’ experiences of resilience were summarized as five main themes and several subthemes. The findings can provide a clear image of the experience of resilience by Iranian adolescents who reside in boarding centers. The two unique aspects of the experiences of the Iranian participants of their life in residential care facilities included “going through life's hardships” and “self-encouragement.” The results of the present study may be beneficial to the design of tools for measuring resilience in Iranian adolescents, as they take the cultural and social conditions of Iran into account; they may also prove helpful in identifying adolescents who need resilience-promoting interventions, or help nurses design their adolescent care programs in a way that targets the promotion of their resilience, thereby playing an important role in the prevention of high-risk behaviors in this vulnerable group. Overall, the results of the present study have helped reveal the different aspects of resilience and provide a new basis for further studies.

## Authors' contributions

MN designed the study, conducted the interviews and analysis and participated in the writing manuscript. FMS and MR engaged in analysis and participated in collaborative dialogue, and revision of manuscript. KNT coordinated the study and participated in collaborative dialogue. AB participated in research design and selected participants.
